# Smoking cessation counseling practices in Jordan: Using the trans-theoretical model

**DOI:** 10.1371/journal.pone.0336111

**Published:** 2025-11-07

**Authors:** Duaa M. Al-Hourani, Rawand A. Khasawneh, Samah F. Al‐Shatnawi, Eman Hammad, Salma Yasser Abu-Saleh

**Affiliations:** 1 Department of Health Care Management and Quality, Faculty of Medicine, Jordan University of Science and Technology, Irbid, Jordan; 2 Department of Clinical Pharmacy, Faculty of Pharmacy, Jordan University of Science and Technology, Irbid, Jordan; 3 Department of Biopharmaceutics and Clinical Pharmacy, Faculty of Pharmacy, University of Jordan, Amman, Jordan; 4 Department of Nutrition and Food Technology Jordan University of Science and Technology, Irbid, Jordan; University of Botswana School of Medicine, BOTSWANA

## Abstract

The trans-theoretical model of behavior change (TTM) is widely used to assess an individual’s readiness to perform the new behavior and categorizes the behavior change into five stages: “pre-contemplation, contemplation, preparation, action, and maintenance.” This study focuses on assessing smoking cessation counseling practices (SCC) among Jordanian healthcare providers (HCPs) across various settings using the TTM. A cross-sectional study was conducted among HCPs (i.e., pharmacists, nurses, physicians, and dentists) working in private and public healthcare settings, using an online self-administered questionnaire. A total of 443 HCPs were included. One-third of HCPs reported asking patients if they smoked at their “first visit only.” Only 24.2% advised every patient to stop smoking, while 17.6% went beyond to assist smokers to make quit attempts, and (16.5%), assessed the willingness of the patients to quit and arrange follow-up quit attempts (10.6%). Only 28.4% of HCPs received training on SCC techniques. The majority of HCPs had a moderate level of confidence in performing SCC practices. HCPs in the private sector were more likely to be active in SCC practices than those in the public sector. Dentists and physicians were more involved in SCC practices than nurses and pharmacists. The study found a significant relationship between HCPs’ stage of change, self-efficacy, and performing SCC practices. This study affords a better understanding of the HCPs’ SCC practices. HCPs are found not to fully perform the “5 As” guidelines in their practices. Future efforts should focus on training and developing education programs that encourage HCPs to perform SCC practice.

## Introduction

Tobacco use stands as a significant and preventable contributor to global mortality and the emergence of diseases. Each year, smoking claims the lives of over eight million individuals, with an additional 1.2 million deaths attributed to exposure to secondhand smoke (SHS) in 2021 alone. The World Health Organization (WHO) has reported that the global tally of active tobacco users has exceeded one billion, and the concerning surge in tobacco consumption is particularly pronounced in the Eastern Mediterranean region [1,2].

According to a recently published national survey, tobacco was reported as a leading risk factor for non-communicable diseases in Jordan and the culprit in nearly 17% of all deaths in 2019 [3,4]. Aside from its well-documented negative effects on health and quality of life, tobacco has a substantial cost, straining the healthcare system and increasing the burden on public funds [3].

Research provides compelling evidence that ceasing smoking can lead to a reduction in smoking-related health issues. Moreover, the concise guidance dispensed by healthcare practitioners to smokers has demonstrated its efficacy as a strategy to enhance efforts to quit smoking, yielding favorable health outcomes [5,6].

Reducing smoking rates stands as a pivotal objective in healthcare, and healthcare providers (HCPs) play a distinct role in aiding smokers’ cessation efforts. Patients perceive physicians as wielding substantial influence over their health choices. Annually, they can reach nearly 80% of all tobacco users, potentially prompting 40% of patients to attempt quitting [7].

It is important to highlight that individuals aspiring to quit smoking are unlikely to succeed without professional counseling and proven cessation medications, which can significantly enhance their chances of successful cessation [8].

In Jordan, smoking cessation support is available in select clinics. Approximately 92% of HCPs in Jordan acknowledge the importance of offering smoking cessation advice. However, a considerable 92.8% of healthcare professionals lack formal training in smoking cessation methodologies to employ with patients, and they do not provide primary prevention services [9,10].

Previous studies in Jordan have primarily focused on smoking prevalence and general cessation knowledge among specific groups of providers, but few have examined behavioral readiness or self-efficacy in counseling practices. To our knowledge, no study has systematically applied the trans-theoretical model (TTM) across multiple categories of healthcare professionals in Jordan.

Therefore, this study was designed to fill this gap by assessing current practices in SCC among HCPs, while also exploring their confidence levels (self-efficacy) and readiness to implement SCC interventions using the TTM. This method makes it possible to comprehend providers’ behavior from a theoretical perspective and highlights opportunities for targeted interventions.

## Methodology

A cross-sectional study was conducted to assess current smoking SCC practices and their associations with stages of the TTM. An online survey targeting HCPs (i.e., physicians, pharmacists, dentists, and nurses) from both public and private healthcare sectors in Jordan was conducted. The sample size was proportionally determined based on the 2020 Ministry of Health report, which recorded 92,511 HCPs (i.e., 30,336 physicians, 32,850 nurses, 19,851 pharmacists, and 9,474 dentists). Using the Raosoft sample size calculator, a minimum sample of 383 participants was calculated based on a 95% confidence level and 5% margin of error. Although this number was statistically sufficient, 443 participants ultimately completed the survey.

A total of 443 healthcare professionals participated in this study, which employed a non-probability convenience sampling method due to its accessibility and feasibility within healthcare settings. Participants were voluntarily recruited through online platforms, such as Facebook and WhatsApp, as well as through direct outreach in practice settings, including hospitals, clinics, and pharmacies. The study employed the TTM of behavior change to assess HCPs’ SCC behavior. TTM has 5 stages (i.e., pre-contemplation, contemplation, preparation, action, maintenance, and relapse) describing gradual behavior change over time [11]. This model aids in understanding and guiding efforts to enhance HCPs’ counseling practices. The association of the two constructs incorporated in TTM (stages of change and self-efficacy) in relation to HCPs’ reported SCC behavior was also assessed. Self-efficacy is a core construct of TTM and social cognitive theory, and it was employed to measure HCPs’ level of confidence in performing SCC interventions.

Pertinent data were collected between January 22nd, 2023 and May 18th, 2023, using a 43-question instrument divided into four parts, developed following a thorough literature review [12–15]. The first part gathered personal and medical information, including age, gender, experience, practice details, smoking status, the number of patients seen per day, prior training in SCC, and interest in developing SCC skills. The second part (reflecting the application of the “5 A’s” model (i.e., ask, advise, assess, assist, arrange). These items assessed frequency, counseling time, and style of smoking intervention practices. The third part included a 5-item scale that determined the SCC behavior stage of change (1–5): pre-contemplation (no intention to perform SCC), contemplation (considering performing SCC within the next 6 months), preparation (considering performing SCC within the next month), action (performing SCC for less than 6 months), and maintenance (performing SCC for more than 6 months). The 5As guideline represents “Ask every patient if they smoke cigarettes,” “Advise every patient to stop smoking cigarettes,” “Assess the patient’s willingness to quit smoking,” “Assist in a quit attempt,” and “Arrange follow-up for smokers following a quit attempt.” The fourth part assessed HCPs’ level of confidence using 13 items on a 5-point Likert scale with options ranging from “certainly not” (1) to “certainly” (5).

The questionnaire was translated into Arabic following a forward–backward translation method to ensure conceptual equivalence. For validity, the instrument was reviewed by three academic experts to establish content validity. This process focused on ensuring that the items were relevant, representative, and comprehensive in addressing the study objectives. A pilot test was also conducted on a small, randomly selected sample of HCPs (*n* = 17) to evaluate the appropriateness, readability, and clarity of the items. The final survey was distributed online in both Arabic and English, based on participant preference. The data collected from the pilot sample were excluded from the final analysis. Feedback from the experts and pilot participants included suggestions for minor phrasing adjustments, which were incorporated into the final version of the instrument.

Cronbach’s alpha coefficient was employed to evaluate the reliability and internal consistency of the study tool, ensuring the precision and dependability of the measurements. The Cronbach’s alpha values for the stage of change and self-efficacy scales were 0.81 and 0.95, respectively. These values were well above the commonly accepted threshold of 0.70, as outlined by George and Mallery, indicating a high degree of reliability [16]. The results suggest that the instrument consistently measured the intended constructs, thereby reinforcing the robustness of the study tool for the targeted population.

The study operationalized the key constructs of the TTM to quantitatively assess HCPs’ SCC behavior. The stages of change were measured using a 5-item scale reflecting participants’ current SCC practices, ranging from pre-contemplation (no intention to provide SCC) to maintenance (providing SCC consistently for more than six months). Self-efficacy was assessed using a 13-item Likert scale measuring participants’ confidence in performing SCC interventions, with scores ranging from 1 (“certainly not confident”) to 5 (“certainly confident”). Each item was adapted from previously validated instruments and reviewed by academic experts for content validity. A pilot test (*n* = 17) confirmed clarity and appropriateness.

Responses for stages of change were categorized according to TTM definitions, while self-efficacy scores were averaged across items. Comparisons of stages and self-efficacy scores were performed across professional categories using appropriate statistical analyses. This operationalization enabled the study to translate theoretical constructs into measurable outcomes and provided a structured framework for understanding providers’ readiness and confidence in delivering SCC interventions.

The study was approved by the Institutional Review Board (IRB) at Jordan University of Science and Technology (Approval Number: 36/155/2023). Participation in the study was voluntary. A letter of informed consent was included within the online survey. The letter included a brief description of the study’s purpose and an explanation that there are no foreseeable risks to participating.

Data were analyzed using Statistical Package for Social Sciences (SPSS) V.25. Descriptive statistics (i.e., frequency, percentage, mean, and standard deviation) were computed as appropriate. One-way analysis of variance was used to compare the stage of change and self-efficacy scores across different healthcare professions, as these were continuous outcomes with more than two groups. Pearson correlation was applied to assess the relationship between self-efficacy, stage of change scores, and continuous demographic variables. A multiple linear regression model was conducted to identify predictors of self-efficacy, adjusting for demographic characteristics. Statistical significance was set at *p* < 0.05. Prior to conducting ANOVA and regression analyses, assumptions of normality were examined, which indicated an approximately normal distribution.

For significant ANOVA results, post-hoc comparisons were performed using Scheffe’s test to identify specific group differences in stage of change scores across responses. These analyses were chosen to appropriately describe associations between variables and differences across groups.

## Results

A total of 443 participants were enrolled, with a mean age of 31.9 ± 8.6 years. In our study, two-thirds of the participants were female (67.7%), and more than half (59%) were married. The majority of the participants had bachelor’s degrees (71.8%). Approximately half of the respondents (51%) reported working in the government sector. Additionally, one-third reported that they were current smokers. The highest percentage of the participants were nurses (34.8%), and 21.8% were MD practitioners (Table 1).

**Table 1:** Demographic Characteristics and Main Variables of Study Participants.

Variables	Mean *SD*	*N* (%)
**Age**	31.9 ± 8.6	–
**Gender**	–	
Female		300 (67.7%)
Male		143 (32.3%)
**Marital status**	–	
Single		166 (37.6%)
Married		261 (59%)
Divorced		15 (3.4%)
**Education level**	–	
Diploma		36 (8.1%)
Bachelor		318 (71.8%)
Master		77 (17.4%)
PhD		12 (2.7%)
**Location of practice**	–	
North		119 (26.9%)
Middle		297 (67%)
South		27 (6.1%)
**Sector of practice**	–	
Governmental		229 (51.6%)
Military		30 (6.8%)
Private		184 (41.5%)
**Healthcare field**		
Medicine		97 (21.9%)
Nursing		154 (34.8%)
Pharmacy		138 (31.2%)
Dentistry		29 (6.5%)
Other		25 (5.6%)
**If you are a doctor, please justify your specialty**		
Internal medicine		56 (12.7%)
Family medicine		6 (1.4%)
General practice		16 (3.6%)
Gynecology		4 (0.9%)
Others		0 (0.0%)
**Type of healthcare setting**		
Public or private health care center		83 (18.7%)
Pharmacy		121 (27.3%)
Hospital setting		239 (53.9%)
**How many years of experience do you have?**		
**Less than one year**		48 (10.8%)
1–5 years		201 (45.4%)
6–10 years		92 (20.8%)
11–15 years		37 (8.4%
16–20 years		37 (8.4%)
More than 20 years		28 (6.3%)
**How many patients do you see each day?**		
Fewer than 10 patients		97 (21.9%)
10–20 patients		138 (31.2%)
21–30 patients		73 (16.5%)
31–40 patients		34 (7.7%)
41–50 patients		28 (6.3%)
More than 50 patients		73 (16.5%)
**How much time do you spend with your patient?**		
Less than 15 min		211 (47.6%)
15–20 min		127 (28.7%)
21–35 min		30 (6.8%)
36–40 min		23 (5.2%)
More than 41 min		52 (11.7%)
**Smoking status**		
Current smoker		139 (31.4%)
Ex-smoker		20 (4.5%)
Non-smoker		284 (64.1%)
**Specify the type of smoking you use**		
Regular cigarettes		66 (14.9%)
E-cigarettes		53 (12%)
Cigar		3 (0.7%)
Hookah		70 (15.8%)
iQOS		9 (2%)
Other		25 (5.6%)
**If you are current smoker, how many cigarettes do you smoke per day? (*N* = 128)**		
Fewer than 10		64 (50%)
11-20		43 (33.6%)
21-40		18 (14.1%)
More than 40		3 (2.3%)
**If you are current smoker, have you ever tried to quit smoking? (*N* = 173)**		
Yes		92 (53.2%)
No		81 (46.8%)
**Total score of Stage Scale**	13.1 ± 6	
**Total score of self-efficacy**	37.9 ± 14.4	

In this study, HCPs demonstrated generally having rather low self-efficacy concerning SCC behaviors. The mean total self-efficacy score was 37.9 (range 13–69). The actual range of scores for the Stage of Change Scale corresponded precisely to the theoretical range (i.e., from 5 to 25). The mean score on the Stage of Change Scale was 13.1 (*SD* ~ 6.01). About one-third of the participants had no intention to do SCC, while another third considered doing this within the next six months. Thus, two-thirds of the respondents had not been performing SCC behaviors routinely for some time.

Moreover, five items were of concern with the participants’ current behaviors regarding smoking cessation interventions. Most of the responding HCPs reported, “I did not arrange follow-up with smoker’s patients,” and “I didn’t assist smoker’s patients,” which would indicate a position about not performing smoking cessation interventions.

### Stage of change in relation to smoking intervention practice behaviors

**Fig 1:**
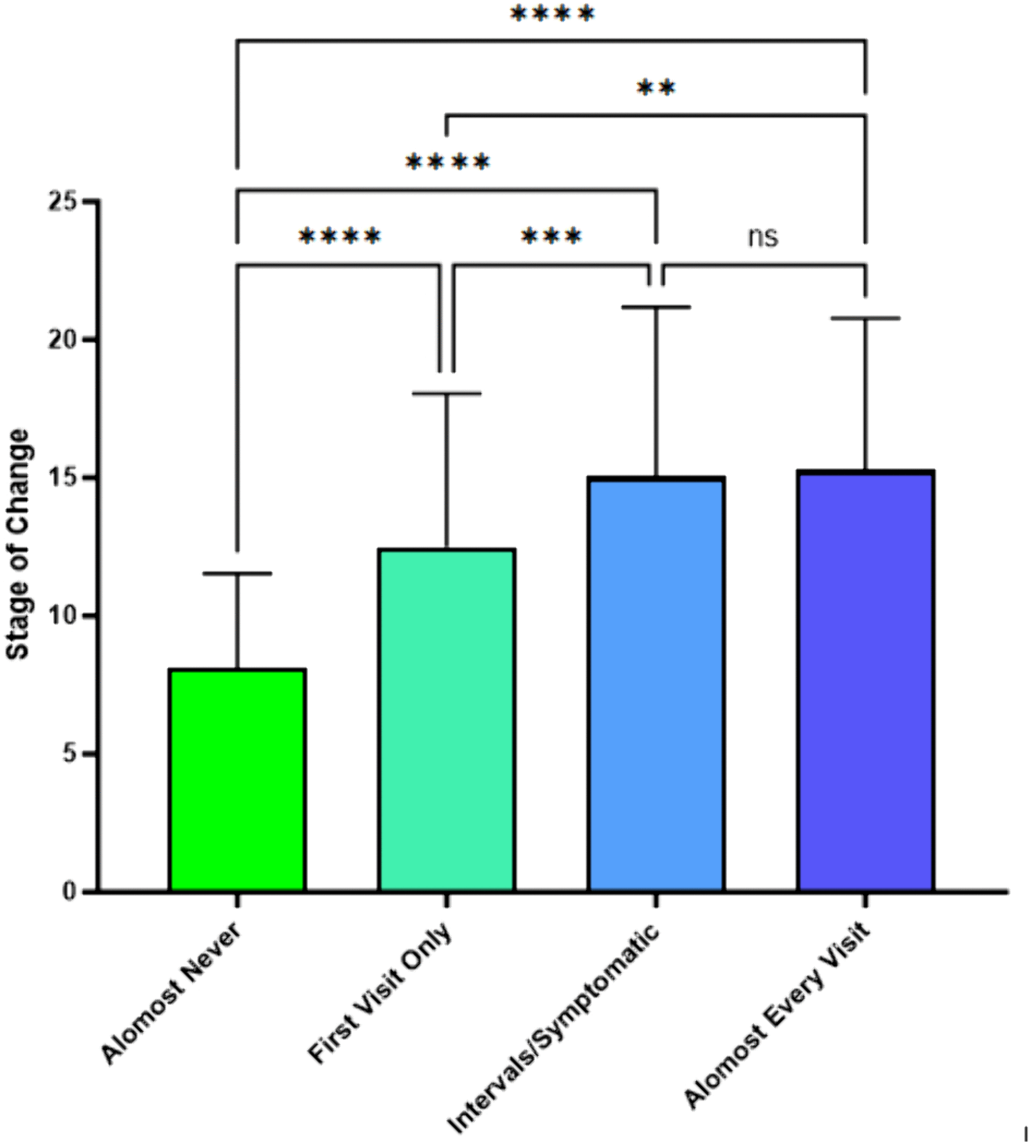
SCC Stage of Change by Self-Reported Frequency of Asking Patients if They Smoke.

The SCC stage of change scores link to responses for five current smoking cessation intervention practice questions were assessed (Fig 1). Stages of change scores related significantly to the first behavior question, “How often do you ask patients if they smoke?” (*F*(3,439) = 32.70, *p* < .001). Mean stage change score for “almost never” ask: 8.13, compared to “first visits only” ask: 12.49, “intervals/when symptomatic” ask: 15.04, and “almost every visit” ask: 15.27.

**Fig 2:**
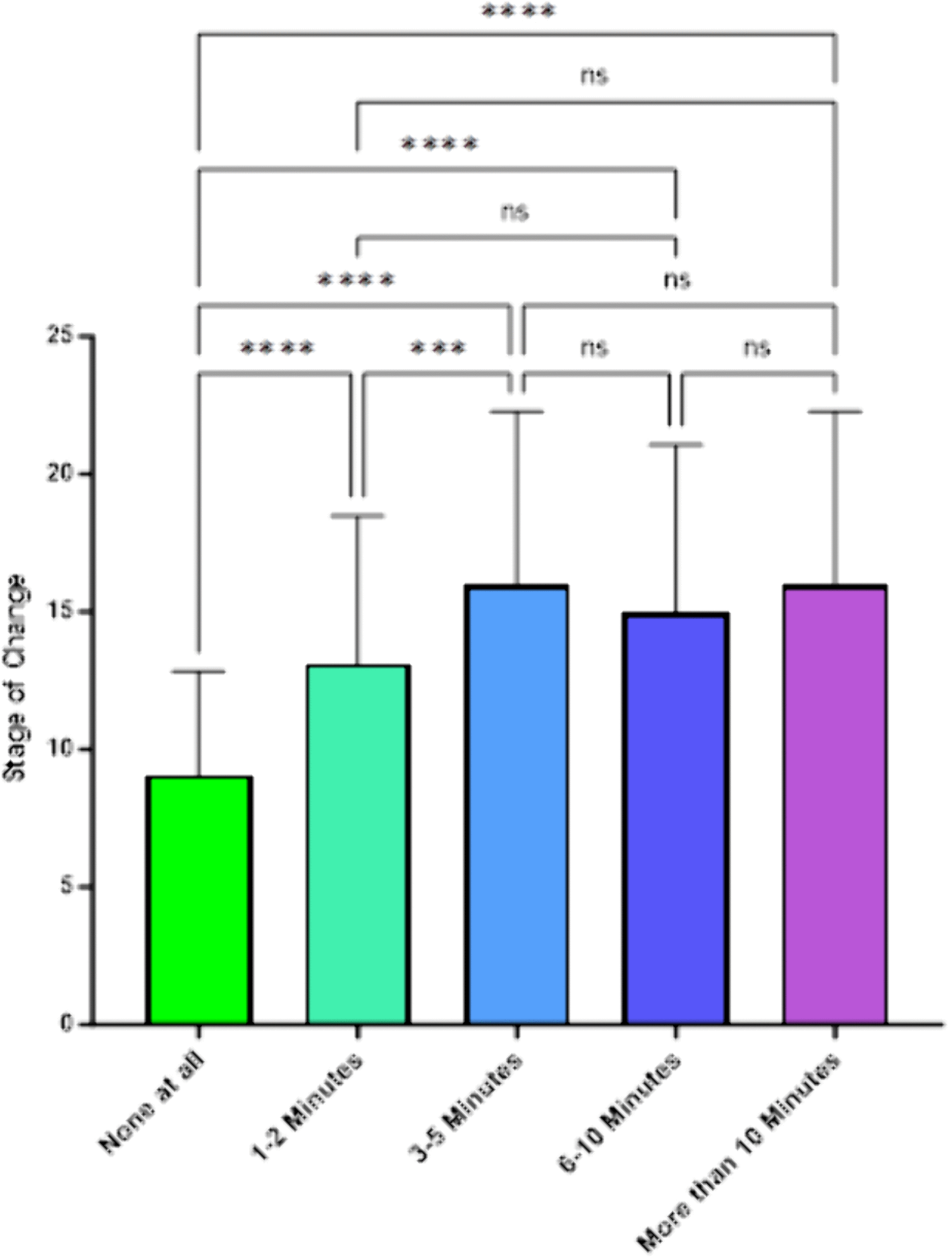
SCC Stage of Change by Self-Reported Amount of Time Spent Talking With Smoking Patients About Their Smoking.

Fig 2 showed a significant link between stage of change scores and the second behavior question, “Time spent discussing smoking?” (*F*(4,438) = 27.33, *p *< .001). The mean stage of change for respondents of “none at all,” “3-5 minutes,” “6-10 minutes,” and “more than 10 minutes” were 9.05, 15.94, 14.97, and 15.95, respectively.

**Fig 3:**
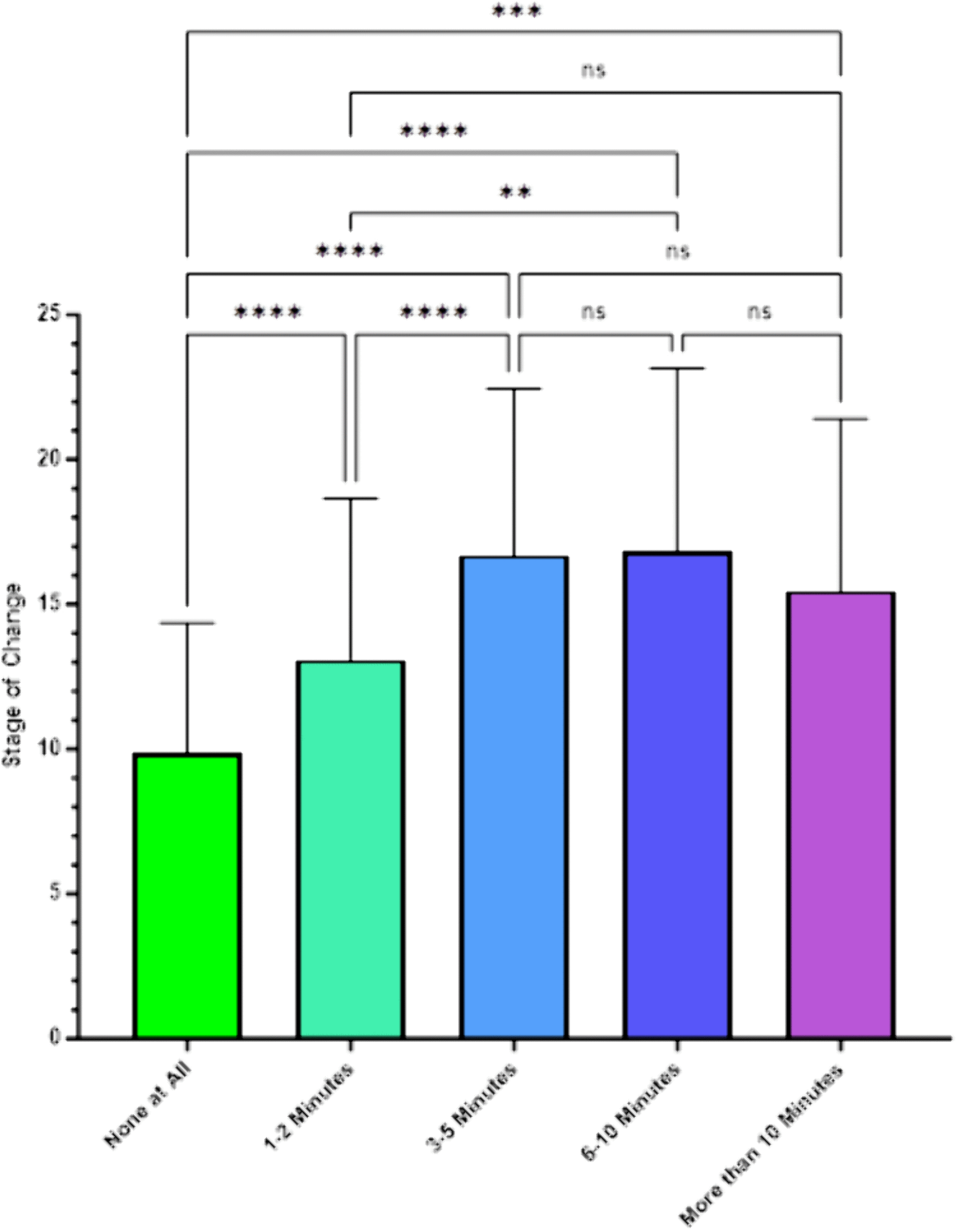
SCC Stage of Change by Amount of Time Spent Assessing Willingness to Make a Smoking Quit Attempt.

Fig 3 presents the results of the analysis of Stage of Change Scale scores by responses to the third question on current smoking intervention practices, “How much time per visit did you spend assessing willingness to make a smoking quit attempt?” The data in the table indicate that this relationship was significant (*F*(4, 438) = 31.72, *p* < .001).

The mean stage of change scores for the groups defined by their responses to this survey question were as follows: among those responding “none at all” (9.88); among those responding “1-2 minutes” (13.03); among those responding “3-5 minutes” (16.6); among those responding “6-10 minutes” (16.8); and among those responding “more than 10 minutes” (15.4). Post-hoc Scheffe contrasts indicated the mean of those who spent no time at all talking about smoking was significantly lower than each of these three group means.

**Fig 4:**
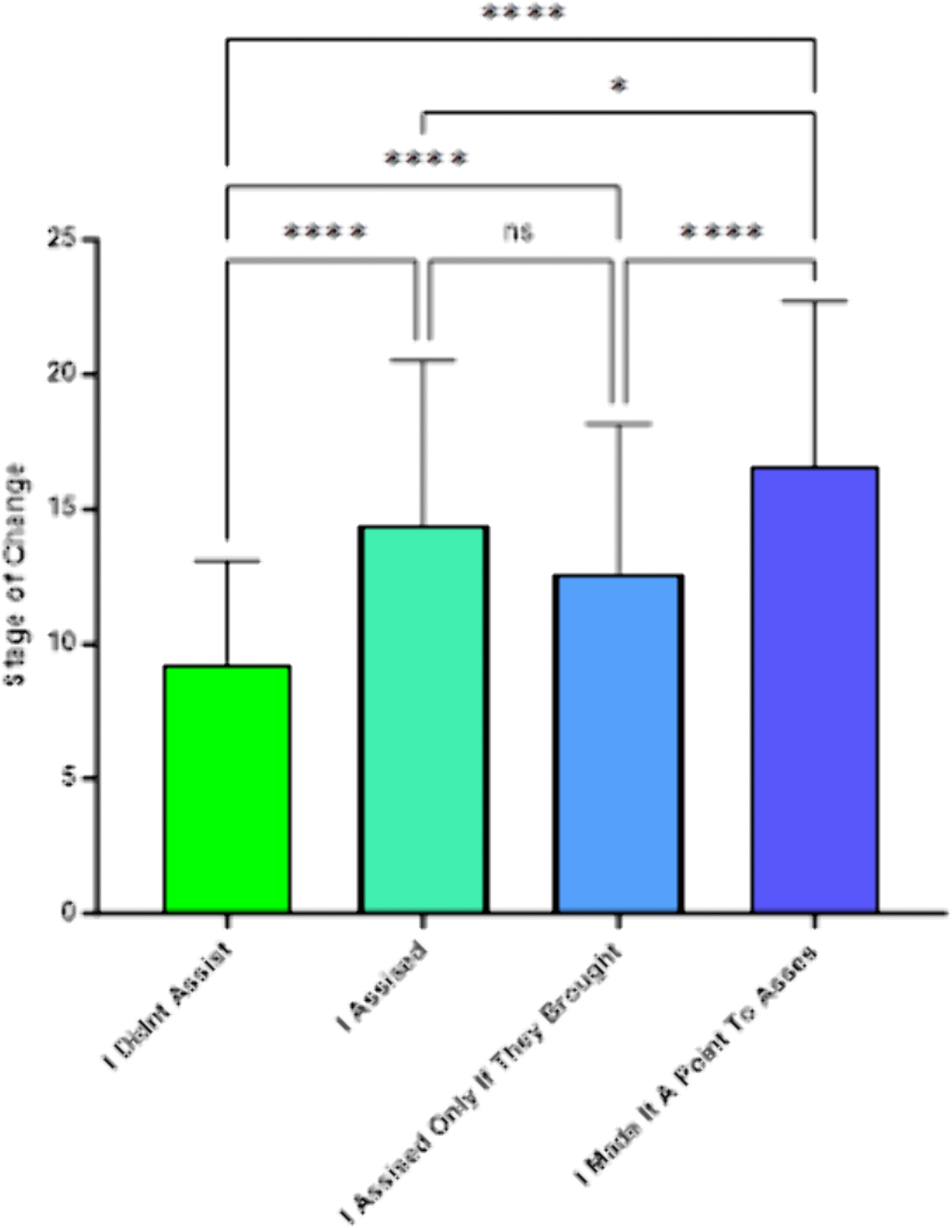
SCC Stage of Change by Responses to the Question: “What Statement Best Describes Your Style of Assisting Patients to Quit?”.

Fig 4 presents the results of the analysis of Stage of Change Scale scores by responses to the fourth question on current smoking intervention practice, “What statement best describes your style of assisting your smoking patients to quit in the last month?” The ANOVA was significant (*F*(3,439) = 33.3, *p* < .001). The mean stage of change score among respondents who said that they did not assist their patients who smoked was 9.29. Among those who responded that “I assisted smokers only if I thought they might be motivated to quit,” the mean Stage of Change Scale score was 14.44, and among those who responded, “I assisted smokers only if they brought up the subject,” the mean was 12.64. One group of respondents had a higher mean on the Stage of Change scale (16.61). These were the respondents who indicated, “I made it a point to assist all patients who smoke regardless of health status or desire to quit.” Post-hoc Scheffe contrasts indicated that the mean of this first group was significantly lower than the mean of any of the other response groups.

**Fig 5:**
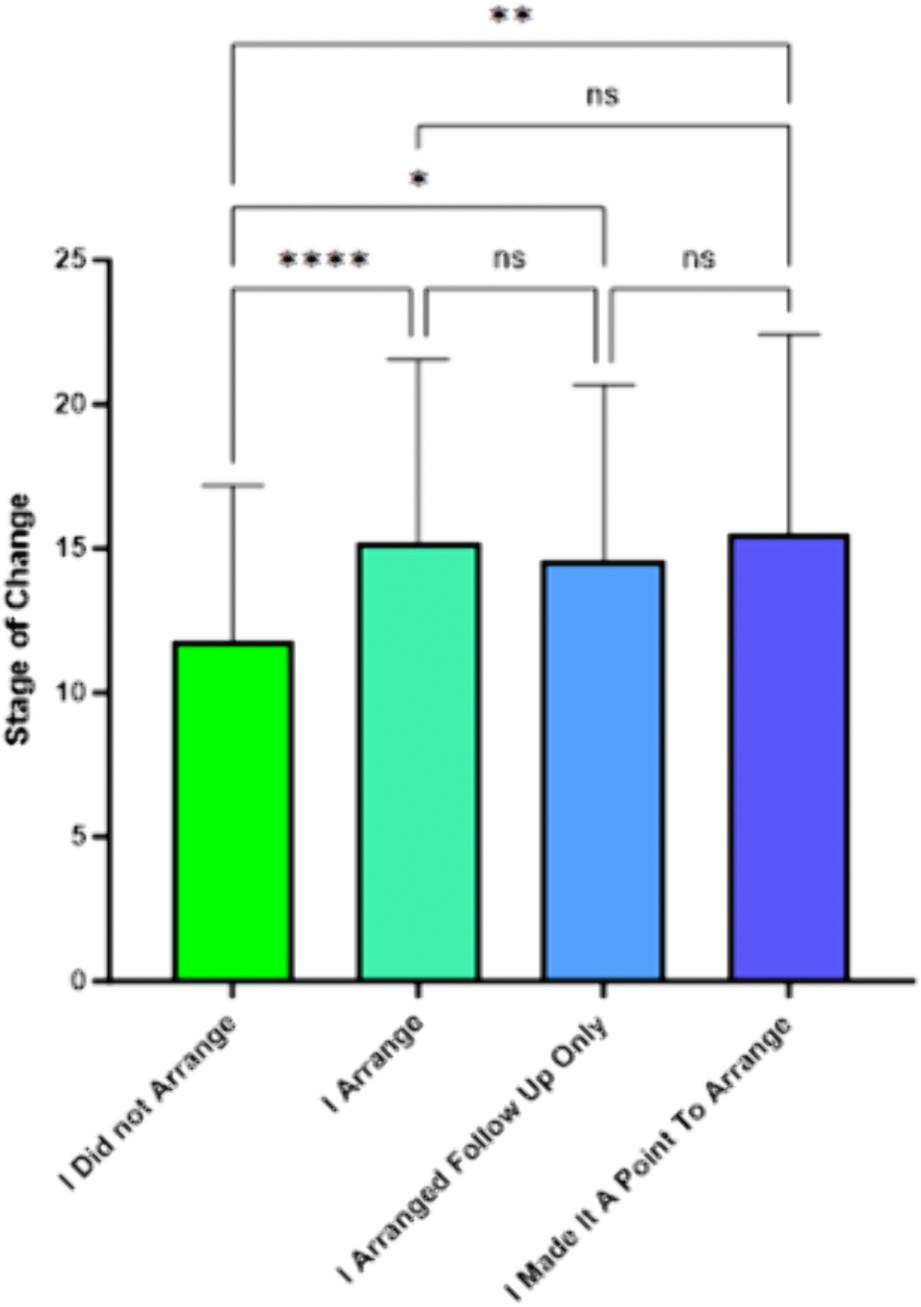
SCC stage of change scores by responses to the question: “What statement best describes your style of arranging follow-up with smokers?”.

The last question on current smoking intervention practice was “What statement best describes your style of arranging follow-up with smokers?” The result indicated ANOVA analysis was significant (*F*(3,439) = 11.55, *p* < .001) among the stages of change by responses to the practice. The mean of the stage of change score among respondents who said they did not arrange follow-ups with patients who smoked was 11.79. Among those who responded, “I arranged follow-up only if I thought the patient might be motivated to quit,” the mean was 15.22. Among those who responded, “I arranged follow-up only if the patient brought up the subject,” the mean was 14.56. Among those responding, “I made it a point to arrange follow-ups with all of my patients who smoke regardless of health status or desire to quit,” the mean was 15.50 (Fig 5). Post-hoc Scheffe contrasts indicated that the mean of this first group was significantly lower than the mean of any of the other response groups.

### Relationship between main variables and demographic characteristics

Pearson correlation was calculated between scores on the self-efficacy scale and scores on the scale measuring the stage of change. This correlation was (*r* = 0.54, *p* < .001). Also, when using a multiple linear regression model to assess the relationship between self-efficacy with stage change score and demographic variables. a significant positive linear relationship was found between total self-efficacy with a total stage of change score (β = 1.23, *ρ*-value < 0.001), age (β = 0.29, *ρ*-value = 0.036), and practice in the private sector (β = 3.3, *ρ*-value = 0.016). The mean was a strong positive relationship between self-efficacy for SCC and the stage of change measure. Those respondents who had high scores on self-efficacy tended to be further along the continuum represented by the stage of change measure. The results of the multiple linear regression analysis are presented in Table 2.

**Table 2:** Relationship between Demographic Characteristics and Stage of Change.

	B	SE	B	ρ-value	CI 95%
Age	0.291	0.138	0.175	0.036	0.019-0.562
Sector of practice(private)	3.35	1.38	0.115	0.016	0.631-6.08
Total stage of change	1.23	0.100	0.519	<0.001	1.037-1.431
Dependent variables on self-efficacy					
	**B**	**SE**	**B**	**ρ-value**	**CI 95%**
Age	0.197	0.096	0.367	0.043	0.006-0.388
Education level (bachelor’s degree)	3.35	1.66	0.199	0.04	0.631-6.08
Total self-efficacy	0.150	0.035	0.393	<0.001	0.080-0.220

Dependent variables on stage of change

## Discussion

This study primarily explored SCC practices among HCPs in Jordan, emphasizing the application of the TTM to assess readiness and self-efficacy. The main findings revealed that only a minority of HCPs consistently implemented all components of the “5 As” guideline, with limited engagement in assessing readiness to quit and arranging follow-ups. Self-efficacy and stage of change were strongly correlated, suggesting that higher confidence levels were associated with more proactive counseling practices.

Although approximately one-third of healthcare professionals (HCPs) in this study reported being current smokers, this finding should be interpreted with caution, as the study was not powered to estimate smoking prevalence. Nevertheless, this proportion appears higher than that previously reported (28.9%) by Matouq et al. [17]. This is concerning, as nearly one in three HCPs smoke, which may negatively influence their likelihood of providing effective smoking cessation counseling (SCC). Evidence indicates that non-smoker HCPs are more likely to advise patients to quit compared to their smoking counterparts [18]. Furthermore, differences in participant composition and methodology limit direct comparisons with prior studies such as Matouq et al., which included no pharmacists or general practitioners and a higher proportion of family physicians and internists.

Only 28.4% of participants received SCC training in our study, similar to Abu-Baker et al., who reported 20.3% of HCPs had training [19]. In Abu-Baker et al., lack of training has been consistently identified as a barrier to SCC and associated with lower self-efficacy [19]. Earlier, Hasan et al. and Marquez et al. linked training with self-efficacy for SCC [10,15].. Untrained HCPs lack primary prevention SCC services. Structured training and professional development are essential to enhance self-efficacy and promote more proactive counseling behaviors among HCPs [10,15,27].

HCPs reported varying practices regarding assessing patients’ smoking status and delivering SCC. Only one-third of HCPs asked patients about smoking, and even fewer assessed willingness to quit or arranged follow-up visits. Compared to a previous study conducted in 2018 by Matouq et al., the results showed that half of the HCPs asked patients about their smoking status and advised them to quit smoking, only 23.7% assessed a willingness to quit, and 17.9% discussed counseling options with smokers. Additionally, only 5.3% of HCPs arranged follow-up sessions for smokers [17]. In our study, a significant proportion of HCPs (60.7%) did not arrange follow-up appointments with patients who smoked.

HCPs play a pivotal role in encouraging healthy behavior in the entire community. Several studies have shown that even brief advice by an HCP has a significant impact on increasing smoking cessation attempts [5,6,20–22].

Aljdani et al. found that most of the HCPs rarely asked their patients about their smoking status [23]. This aligns with our finding that only 26.60% of HCPs consistently asked every patient if they smoked.

Approximately 24.2% of HCPs advised patients to stop smoking. This is inconsistent with previous studies that reported that approximately half of the HCPs asked patients about their smoking status and advised them to quit smoking [17,19]. Aside from this, and congruent with other studies, our results indicate that HCPs generally provide limited assistance in smoking cessation, with only a small proportion assessing willingness to quit and arranging follow-up visits or referrals to cessation treatments [17,19].

The result showed that the majority of HCPs were in the contemplation stage of change, indicating they planned to implement SCC within the next 6 months. This suggests a potential area for improvement, as a significant portion of HCPs may not yet fully prioritize or implement “5As” guidelines to support smokers in smoking cessation attempts.

Significant differences were found among HCPs regarding their stage of change and self-efficacy in providing the “5As” intervention. Dentists demonstrated higher scores for stage of change and more consistent implementation of the 5 A’s compared to the other professions. This finding is consistent with a previous study by Al-Maweri et al., who reported that most dentists frequently asked patients about their smoking status and advised them to quit due to their knowledge of identifying the harmful effects of smoking on oral health and helping patients in quitting smoking during regular check-ups [24].

However, Matouq et al. reported contradictory results showing that physicians were more committed to implementing “5As” SCC interventions and assisted more patients in quitting attempts compared to nurses or dentists [17]. In contrast, our findings found that dental professionals demonstrated a higher level of readiness and confidence to participate in SCC with their patients compared to other HCPs’ professions.

Furthermore, this study highlights the link between stage of change and effective smoking counseling, aligning with past TTM research [12,14,25,26]. Park et al. emphasized the importance of considering the stage of change when designing interventions [25]. Our findings underscore the relevance of incorporating stage-specific strategies and targeted training to enhance HCPs’ SCC practices. Overall, HCPs in this study exhibited moderate confidence in delivering SCC, with the highest confidence observed in asking patients about smoking, advising quitting, providing brief counseling, and prescribing medication.

Karacabeyli et al. reported that insufficient training led to low counseling confidence [27]. Similarly, Hasan et al. found that limited self-efficacy hindered effective counseling, but noted that targeted interventions improved HCPs’ confidence in delivering the 5 A’s [15]. Collectively, prior studies have emphasized the importance of structured training programs to enhance self-efficacy and intent for tobacco cessation [15,27].

The study found that higher self-efficacy scores were associated with more proactive counseling behavior, including asking more questions, conducting longer discussions, and thoroughly assessing patients’ willingness to quit. Also, proactive HCPs who assisted all smoking patients, regardless of motivation or health status, demonstrate higher self-efficacy scores. Likewise, Elshatarat et al. emphasized the critical role of self-efficacy in promoting behavior change and effective SCC practices [28]. Conversely, low self-efficacy levels among healthcare workers were shown to hinder their ability to provide effective counseling, as reported by Garg et al. [29]. Previous studies indicated that self-efficacy level is influenced by prior training and education [19,29–31]. Moreover, Hudmon et al. proved that higher self-efficacy is linked to increased frequency of smoking counseling practices [32]. In 2020, a quasi-experimental study in Thailand employing a pre-test and post-test design involving nurses showed that targeted training interventions enhanced both knowledge and level of confidence in providing SCC [33]. These findings collectively suggest that comprehensive training programs can strengthen HCPs’ self-efficacy and promote effective SCC practices.

This study demonstrated a positive relationship between SCC self-efficacy and the stage of change, operationalized according to the “5A’s” of the brief SCC guideline. Significant positive linear relationships were also observed between total self-efficacy scores and stage of change, as well as age, level of education, and practice in the private sector. Prior research has shown that physicians are more likely to provide counseling when confident in their abilities [25]. Zapka et al. found action-stage physicians with high self-efficacy performed better counseling than those in the pre-contemplation stage, highlighting that increased self-efficacy improves HCP SCC practices [26].

These results supported the role of self-efficacy in advancing progression through the stages of change [34–39]. Additionally, our findings align with Garg et al.’s linking higher education to greater self-efficacy and with Elkhadragy et al., who emphasized the influence of self-efficacy on SCC intentions. Overall, these findings underscore the importance of education and training to enhance self-efficacy for better SCC delivery [29,40].

Our findings highlight the need for policy interventions to integrate SCC into routine care and support HCPs in progressing through the stages of change. These findings have significant consequences for Jordanian health policy and clinical practice. The limited number of HCPs trained in SCC underscores the necessity for organized, profession-specific educational programs to improve knowledge, skills, and self-efficacy. Integrating SCC into standard clinical protocols and offering ongoing professional development can enhance compliance with the “5 A’s” guidelines. Additionally, strategies to support HCPs who smoke in quitting themselves may increase their likelihood of advising patients. Strengthening self-efficacy through targeted training and mentorship is essential to promote proactive counseling and improve patient outcomes in smoking cessation initiatives.

### Study implications

The findings of this study have significant implications for healthcare professionals and health authorities in Jordan. This research highlights current SCC behaviors and practices among HCPs, identifies areas for improvement, and underscores the relevance of the TTM in guiding behavior change at different stages of readiness. Incorporation of the TTM strengthens the study and supports the development of evidence-based interventions, serving as a benchmark for future research and facilitating comparisons with similar studies in other regions.

To translate these findings into actionable strategies, future training and educational programs should be structured and targeted. Specific approaches may include interactive workshops with role-playing and case simulations, integration of SCC practices and brief advice into educational curricula and job descriptions, ongoing evaluation of HCP performance post-training, and the use of “train-the-trainer” models to expand program reach across healthcare facilities. These interventions focus on enhancing self-efficacy, improving adherence to the “5 A’s” guideline, and strengthening the delivery of SCC, ultimately contributing to reducing smoking prevalence and improving population health outcomes in Jordan.

One key strength of this study is that it is the first to evaluate HCPs’ SCC stage of change and self-efficacy in applying the “5 A’s” guideline in Jordan, using the TTM framework. This study has several limitations. First, the cross-sectional design restricts the ability to draw causal inferences regarding the relationships between self-efficacy, stage of change, and SCC practices. Second, data were self-reported, introducing potential reporting and social desirability bias that could affect the reliability of the findings. Thirdly, while the sample size is robust, the use of a convenience sampling approach and online recruitment may still limit the generalizability of the findings, as certain subgroups of healthcare professionals could be underrepresented. Response bias also remains a potential concern. Additionally, to gain a deeper understanding of factors influencing engagement with SCC interventions, future research employing qualitative methods would be valuable, providing insights into motivators, barriers, and individual capacities. Longitudinal studies are also needed to evaluate the causal impact of SCC practices over time.

## Supporting information

S1 Data Raw Data. (XLSX)
